# Muscle sodium content in patients with Myalgic Encephalomyelitis/Chronic Fatigue Syndrome

**DOI:** 10.1186/s12967-022-03616-z

**Published:** 2022-12-09

**Authors:** Elisabeth Petter, Carmen Scheibenbogen, Peter Linz, Christian Stehning, Klaus Wirth, Titus Kuehne, Marcus Kelm

**Affiliations:** 1 Institute of Medical Immunology, Charité Universitätsmedizin Berlin, Campus Virchow, Berlin, Germany; 2 Institute of Computer-Assisted Cardiovascular Medicine, Charité Universitätsmedizin Berlin, Berlin, Germany; 3 Department of Congenital Heart Disease, German Heart Center Berlin, Berlin, Germany; 4 Institute of Radiology, Friedrich‐Alexander‐Universität Erlangen‐Nürnberg (FAU), University Hospital Erlangen, Erlangen, Germany; 5 Philips Healthcare, Hamburg, Germany; 6 Institute of General Pharmacology and Toxicology, University Hospital Frankfurt am Main, Goethe-University, Theodor-Stern Kai 7, Frankfurt am Main, Germany; 7 Berlin Institute of Health (BIH), Berlin, Germany; 8 German Centre for Cardiovascular Research (DZHK), Partner Site, Berlin, Germany

**Keywords:** Chronic Fatigue Syndrome, Sodium, Magnetic Resonance Imaging, Muscle, skeletal, Myalgia, Exercise, Sodium–potassium-exchanging ATPase, Sodium–hydrogen exchangers

## Abstract

**Background:**

Muscle fatigue and pain are key symptoms of Myalgic Encephalomyelitis/Chronic Fatigue Syndrome (ME/CFS). Although the pathophysiology is not yet fully understood, there is ample evidence for hypoperfusion which may result in electrolyte imbalance and sodium overload in muscles. Therefore, the aim of this study was to assess levels of sodium content in muscles of patients with ME/CFS and to compare these to healthy controls.

**Methods:**

Six female patients with ME/CFS and six age, BMI and sex matched controls underwent ^23^Na-MRI of the left lower leg using a clinical 3T MR scanner before and after 3 min of plantar flexion exercise. Sodium reference phantoms with solutions of 10, 20, 30 and 40 mmol/L NaCl were used for quantification. Muscle sodium content over 40 min was measured using a dedicated plugin in the open-source DICOM viewer Horos. Handgrip strength was measured and correlated with sodium content.

**Results:**

Baseline tissue sodium content was higher in all 5 lower leg muscle compartments in ME/CFS compared to controls. Within the anterior extensor muscle compartment, the highest difference in baseline muscle sodium content between ME/CFS and controls was found (mean ± SD; 12.20 ± 1.66 mM in ME/CFS versus 9.38 ± 0.71 mM in controls, p = 0.0034). Directly after exercise, tissue sodium content increased in gastrocnemius and triceps surae muscles with + 30% in ME/CFS (p = 0.0005) and + 24% in controls (p = 0.0007) in the medial gastrocnemius muscle but not in the extensor muscles which were not exercised. Compared to baseline, the increase of sodium content in medial gastrocnemius muscle was stronger in ME/CFS than in controls with + 30% versus + 17% to baseline at 12 min (p = 0.0326) and + 29% versus + 16% to baseline at 15 min (p = 0.0265). Patients had reduced average handgrip strength which was associated with increased average muscle tissue sodium content (p = 0.0319, R^2^ = 0.3832).

**Conclusion:**

Muscle sodium content before and after exercise was higher in ME/CFS than in healthy controls. Furthermore, our findings indicate an inverse correlation between muscle sodium content and handgrip strength. These findings provide evidence that sodium overload may play a role in the pathophysiology of ME/CFS and may allow for potential therapeutic targeting.

**Supplementary Information:**

The online version contains supplementary material available at 10.1186/s12967-022-03616-z.

## Background

Myalgic Encephalomyelitis/Chronic Fatigue Syndrome (ME/CFS) is a complex and chronic disease with a worldwide prevalence of up to 0.9% often triggered by viral infections such as EBV or SARS-Cov2 [1, 2]. Patients suffer from severe central and muscular fatigue, sleep disturbance, cognitive impairment, and immune and autonomic dysfunction. The cardinal symptom is exertional intolerance with post-exertional malaise, which describes a disproportionate aggravation of symptoms and a prolonged recovery period after physical or mental exertion [3]. Muscle fatigue and myalgia are key symptoms of ME/CFS. Muscle fatigue and fatigability can be measured by the assessment of hand grip strength [4]. Although the etiology and pathophysiology of ME/CFS is not fully understood yet, there is ample evidence for an autoantibody mediated dysregulation of the autonomic nervous system and disturbed vascular regulation [5–8]. Endothelial dysfunction, hypoperfusion of muscles and impaired cerebral blood flow are assumed to be key mechanisms for symptoms like fatigue, myalgia, post-exertional malaise and impaired cognition [9, 10]. Patients with ME/CFS performed worse than healthy controls in a controlled repeated exercise study and showed increased intramuscular acidosis and abnormalities in recovery of muscular pH after standardized exercise of lower leg muscles [11, 12]. Compared to healthy controls, diminished proton efflux was observed in ME/CFS patients immediately after exercise and maximum proton efflux was also reduced [13]. Disturbances of ions in skeletal muscles such as sodium overload and subsequent calcium overload was hypothesized as consequences of hypoxemia in ME/CFS [14]. Based on these assumptions, in this study we analyzed sodium content in skeletal muscle tissue in patients with ME/CFS.

Recent studies have established ^23^Na-magnetic resonance imaging (MRI) as a reliable, non-invasive method to quantify sodium content in muscle tissue [15–17]. The method has been used in a broad set of study cohorts, such as arterial hypertension [18], acute heart failure [19], kidney failure [20], diabetes mellitus [21], multiple sclerosis [22] and muscle diseases [23, 24]. Duchenne muscular dystrophy and muscle channelopathy were associated with elevated tissue sodium [25, 26]. Some researchers already conducted sodium MRI studies with exercise or muscle strain [27, 28]. Increased levels of sodium content in exercised muscles were found after leg strain compared to baseline in healthy subjects [29, 30]. Therefore, the aim of this study was to comparatively assess muscle sodium in ME/CFS and healthy controls at baseline and after exercise.

## Materials and methods

### Subjects and study design

In this pilot study, six patients with ME/CFS and six healthy controls were studied. All patients were recruited between August 2020 and November 2020 at the Institute of Medical Immunology at Charité where they were previously diagnosed with ME/CFS based on Canadian Consensus Criteria. Inclusion Criteria were: ME/CFS triggered by an infection, muscle pain > 5 assessed by a likert scale from 0 (none) to 10 (severest), a Bell Score from 20 to 40 [1, 31, 32], female sex (as women suffer more often from ME/CFS) and age 20–45 years. Healthy controls were age, BMI and sex matched with no history of illnesses and no medication, that could affect the muscle function. They were required to have sedentary jobs and perform less than three hours of physical activity per week [33].

### Exercise protocol

Study participants were instructed not to exercise or perform intense leg movements for 1 week before the MR examination. On examination day, patients were picked up from home by taxi and healthy controls used public transportation or car to not overstress their muscles. Before the subjects´ left calf was scanned at its widest circumference, they rested in a lying position for at least 30 min to reach a constant distribution of the interstitial volume and to ensure comparable resting states of the muscles [34]. After the initial baseline imaging subjects were asked to get off the MR to perform heel raises to exercise the triceps surae muscle. The exercise protocol contained 3 min of anaerobic dynamic training [27]. Plantar flexion was done by raising both heels from standing position, stand on tiptoes and return to the floor, against one´s own body weight with a frequency of 30/min [13, 29, 30]. Subjects were repositioned to MR scanner immediately after exercise for the second run of the measurement protocol. To assess muscular fatigue, handgrip strength was measured in all study participants with an electric dynamometer (CAMRY, model: SCACAM-EH101) in two separate sessions. Patients and controls had to sit in an upright position and place the forearm of the dominant hand on a standard table in full supination. The handle was pulled 10 times with maximum force for 3 s, each time followed by a 5 s relaxation phase [35, 36]. A second session was performed after a recovery break of 60 min. Heart rate and blood pressure were measured alongside to image acquisition before and after exertion.

### MRI acquisition protocol and data processing

Imaging was performed on a clinical 3 Tesla MR scanner (Philips Ingenia, Software Release 5.6.1, Philips Healthcare, Best, The Netherlands) with a ^23^Na send/receive knee-coil (Rapid Biomedical, Rimpar, Germany) using a 2D spoiled gradient multi-echo sequence with flyback gradients (12 echoes, echo times TE = 2.44 ms + n × 3.05 ms), repetition time TR = 100 ms, flip angle FA = 90°, measured resolution = 3 × 3 × 30 mm^2^, 132 averages, scan duration TA = 14 min. To assess dynamic alterations of sodium levels during the prolonged imaging procedure, complex images were stored for each individual average, in order to allow for retrospective averaging with smaller temporal footprints. Averaging was performed via a dedicated postprocessing plugin for Horos, to enable complex averaging from a user-defined subset of averages and echoes. The ^23^Na signal was averaged over the first three echoes of the multi-echo acquisition, acquired at TE_n_ = 2.44 ms, 5.49 ms and 8.54 ms, restricting the visualization to long T2* sodium components. Furthermore, a moving average filter over 80 respective average was employed, yielding a time-resolved reconstruction with 53 timeframes over the prolonged acquisition duration. Along with the subjects´ calf, four calibration phantoms containing aqueous solutions of 10, 20, 30 and 40 mmol/L (mM) NaCl were scanned as reference standards [18]. Tissue water content was measured by 1H-MRI, using a fat-saturated inversion-prepared SE sequence (inversion time, TI = 210 ms; TA = 6.27 min; TE = 12 ms; TR = 3000 ms; FA = 90◦; 1 average, resolution: 1.5 × 1.5 × 5 mm^3^), as already conducted by other scientists [19, 37].

**Fig. 1:**
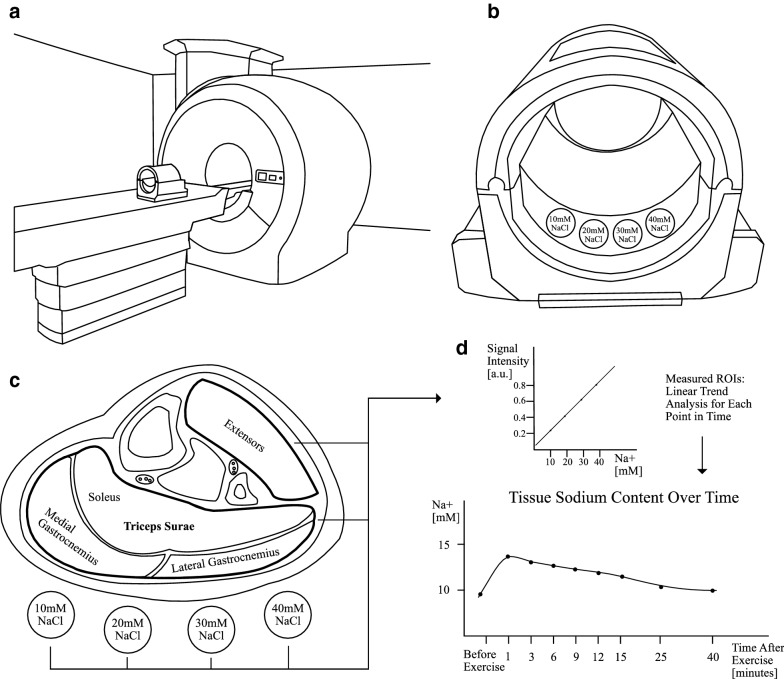
Schematic overview of methods. **a** Clinical 3 Tesla MR scanner with ^23^Na knee-coil for lower leg muscles placed in. **b** ^23^Na knee-coil with four calibration phantoms containing solutions of 10, 20, 30 and 40 mmol/L NaCl. **c** After dynamic reconstruction of the data based on a dedicated plugin, regions of interest (ROI) were drawn in Horos on MR images for all lower leg muscles and all calibration phantoms. **d** Signal intensities of ROIs (measured in arbitrary units) were translated to sodium content in mM using a linear trend analysis. Finally, sodium content of muscle tissue was assessed over time after exercise

Regions of interest (ROI) were drawn using the medical image viewer Horos (version 3.3.6) and the anatomical image (T1-weighted spoiled gradient echo sequence) as guideline. ROIs were drawn over noise background (0 mM NaCl) and over all reference phantoms and were measured in arbitrary units (a.u.). The respective signal intensities served as calibration standards by translating intensity to concentration in a linear trend analysis. Important ROIs enclosed (a) the largest lower leg muscle (triceps surae consisting of the medial and lateral gastrocnemius and soleus muscle) and (b) the extensor muscles of the anterior compartment of the leg (tibialis anterior, extensor hallucis, extensor digitorum and fibularis muscles), which were not exercised were used for internal control. Since prominent vascular structures are rich in sodium, they were excluded during measurement. The primary outcome was defined as the absolute sodium content in the muscle tissue. A graphical summary of the study methods is outlined in Fig. 1.

### Statistical analysis

Continuous data are presented as means with standard deviation (± SD), unless stated otherwise. Using the Shapiro–Wilk test and Kolmogorov–Smirnov test, data distribution was tested. The 95% confidence intervals were calculated and p < 0.05 was considered significant for all statistical tests. P-values are uncorrected and considered descriptive due to multiple testing. Paired t-test was used within the groups to test the progression of sodium and water content in muscle tissue over time, and to compare the two sessions of handgrip strength measurement. Unpaired t-test was used to test the differences in tissue sodium and water content, characteristics, and handgrip strength between patients with ME/CFS and controls. Differences in decrease of sodium content after its peak values were assessed by comparison of linear regression lines, which is one use of ANCOVA. Correlations were assessed by linear regression model and Pearson correlation coefficient. GraphPad Prism (version 9.0.2) was used for statistical analysis.

## Results

### Study population

Muscle sodium and water content at baseline and changes over time after lower leg muscle exercise were measured noninvasively and analyzed in a total of 12 study subjects. Six patients with ME/CFS were compared to six matched healthy controls, the characteristics of the study population are shown in Table 1. Age, BMI, and sex did not differ between the groups, neither did behaviors regarding salt appetite, salty food craving and consumption of beverages. All study subjects had an increase in heart rate and blood pressure directly after exercise. All subjects reported mild pain and exhaustion immediately after exercise. Controls had painful sore muscles for two days after exercise. All patients with ME/CFS reported post-exertional malaise following exercise.

**Table 1:** Baseline characteristics

Subjects	Patients with ME/CFS (n = 6)	Controls (n = 6)	P-value ME/CFS versus Controls
Sex	Female	Female	–
Age (years)	30.3 (7.6)	27.3 (4.8)	0.4308
BMI (kg/m^2^)	21.6 (4.1)	23.4 (5.1)	0.5253
Bell Score [0–100]	30 (6.3)	–	–
Chalder Fatigue Score [0–33]	29.5 (1.4)	–	–
Systolic blood pressure baseline	108.0 (11.1)	115.7 (8.0)	0.1997
Diastolic blood pressure baseline	63.2 (7.5)	62.0 (2.8)	0.7295
Mean arterial pressure baseline	77.3 (8.8)	78.7 (5.1)	0.7560
Heart rate baseline	63.8 (7.9)	64.7 (6.0)	0.8411
Systolic blood pressure after exercise	119.8 (12.0)	125.5 (10.8)	0.4116
Diastolic blood pressure after exercise	72.2 (11.9)	71.0 (3.3)	0.8215
Mean arterial pressure after exercise	90.7 (8.4)	87 (4.9)	0.3777
Heart rate after exercise	76.5 (6.7)	69.8 (4.2)	0.0672
*Salt questionnaire* [37]			
“How much do you like salty food?”	6.0 (0.9)	5.7 (2.9)	0.7961
“How often do you add more salt to your food?”	3.7 (2.0)	4.7 (3.8)	0.5819
“How much do you like salty snacks such as crisps?”	5.5 (1.4)	5.3 (3.4)	0.9146
“How often do you eat in fast food restaurants?”	2.0 (1.5)	2.3 (0.8)	0.6510
“How much money do you usually spend there?” (€)	3.8 (4.9)	7.0 (4.3)	0.2640
“How often do you drink beverages without additional flavour?”	7.7 (2.7)	6.2 (3.7)	0.4438
“How much do you drink daily?” (L)	2.1 (0.4)	2.1 (0.4)	> 0.9999

ME/CFS, Myalgic Encephalomyelitis/Chronic Fatigue Syndrome. Mean values with standard deviation (SD) in brackets, unless stated otherwise. Questionnaire on a scale from 1–10 (1 = I strongly agree; 10 = I strongly disagree). P value refers to comparison of both groups

### Dynamic of sodium and water content

**Fig. 2:**
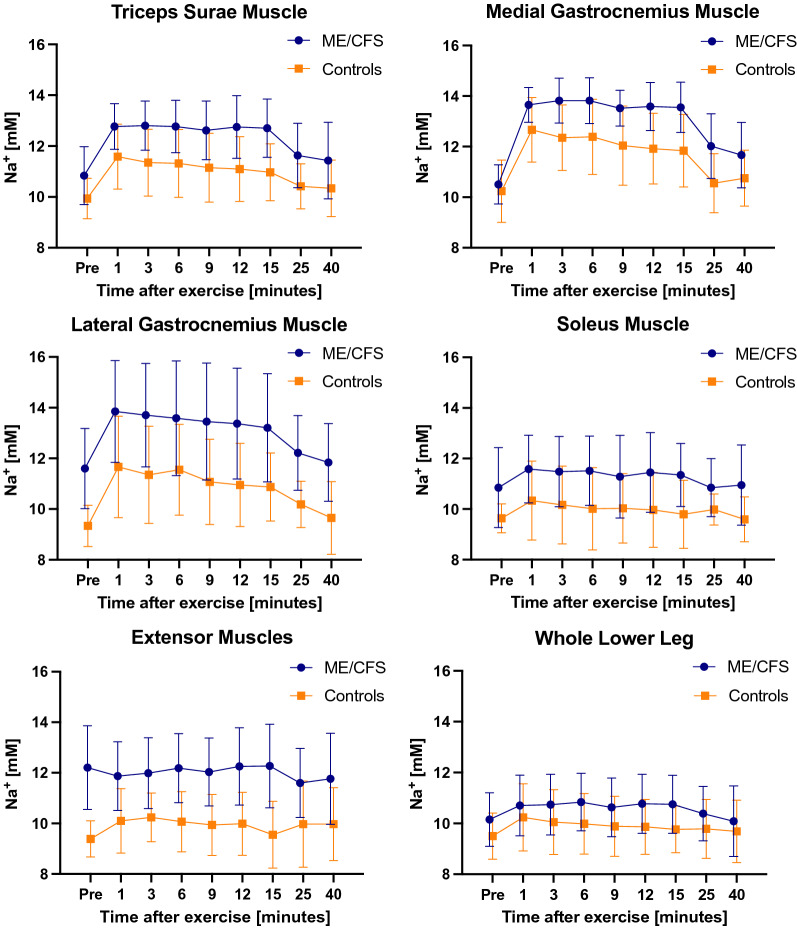
Mean tissue sodium content in lower leg muscles. Mean values in mM with standard deviation. Curves were plotted for six compartments of the lower leg of patients with ME/CFS and controls: triceps surae, medial gastrocnemius, lateral gastrocnemius, soleus, extensor muscles and whole lower leg. After exercise, tissue sodium content increased in all exercised muscle compartments in both groups. Patients showed higher tissue sodium content in exercised muscles compared to controls. Tissue sodium content decreased within the 40-min recovery period in both groups. In extensor muscles which were not exercised, patients showed continuously higher values than controls

**Fig. 3:**
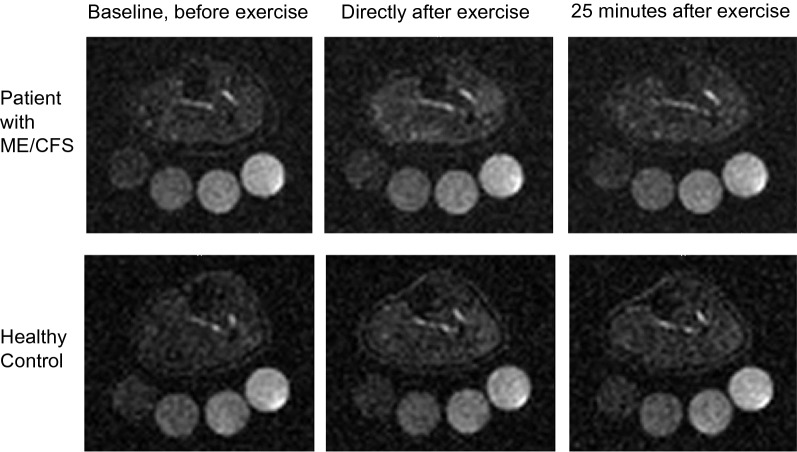
^23^Na MR images of the lower leg of a ME/CFS patient and a healthy control. Increased sodium signals are visible in the muscle tissue directly after exercise in comparison to baseline and after recovery period of 25 min. The reference phantoms below the leg appear in variable intensity due to their sodium concentrations (from left to right: 10, 20, 30 and 40 mmol/L NaCl)

Image quality was satisfactory for all 12 study subjects and none of the images had remarkable motion artefacts. Dynamic progressions of tissue sodium content in lower leg muscles over time in patients and in controls are shown in Fig. 2. Directly after exercise, we found elevated sodium content in triceps surae, medial and lateral gastrocnemius. The strongest increase of tissue sodium content was observed in medial gastrocnemius muscle (patients from 10.50 ± 0.78 to 13.65 ± 0.69 at minute 1, + 30%, p = 0.0005; controls from 10.23 ± 1.24 to 12.67 ± 1.28 at minute 1, + 24%, p = 0.0007) as shown in Fig. 2. Within the 40-min recovery period, sodium content decreased in these muscles. Neither ME/CFS patients nor controls had an increase upon exertion in sodium content in the extensor muscles and only a minor increase in the soleus, which were not involved in the exercise. There were no changes in tissue water content in any muscles of the lower leg in both groups. Values of tissue sodium and water content of different muscle compartments are outlined in Table 2. Figure 3 shows representative ^23^Na images from two subjects at baseline, directly after exercise and 25 min after exercise. The increased intensities which are faintly visible in the images directly after exercise correlate with an increase of tissue sodium content measured and expressed in mM.

**Table 2:** Tissue sodium content and tissue water content

	ME/CFS					Controls				
	Baseline		Directly after exercise		40 minutes after exercise	Baseline		Directly after exercise		40 minutes after exercise
Na^+^ [mM]										
Triceps surae										
Mean (SD)	10.83 (1.15)		12.77 (0.90)		11.43 (1.51)	9.93 (0.79)		11.58 (1.13)		10.33 (1.12)
∆ (%)		+18		− 10			+17		− 11	
P value		<0.0001		0.0090			0.0081		0.0002	
Medial gastrocnemius										
Mean (SD)	10.50 (0.78)		13.65(0.69)		11.67 (1.30)	10.23 (1.24)		12.67 (1.28)		10.75 (1.11)
∆ (%)		+30		− 15			+24		− 15	
P value		0.0005		0.0182			0.0007		0.0002	
Lateral gastrocnemius										
Mean (SD)	11.60 (1.58)		13.85 (2.01)		11.83 (1.54)	9.33 (0.82)		11.67 (2.01)		9.65 (1.44)
∆ (%)		+19		− 15			+25		− 17	
P value		0.0060		0.0023			0.0373		0.0099	
Soleus										
Mean (SD)	10.85 (1.58)		11.58 (1.34)		10.95 (1.59)	9.63 (0.58)		10.33 (1.56)		9.60 (0.89)
∆ (%)		+7		− 5			+7		− 7	
P value		0.0197		0.1334			0.3220		0.0974	
Extensors										
Mean (SD)	12.20 (1.66)		11.87 (1.37)		11.77 (1.81)	9.38 (0.71)		10.10 (1.28)		9.97 (1.45)
∆ (%)		− 3		− 1			+8		− 1	
P value		0.5354		0.9014			0.2492		0.7796	
H_2_O [kg/L]										
Triceps surae										
Mean (SD)	1.16 (0.09)		1.16 (0.06)		–	1.16(0.07)		1.16 (0.07)		–
∆ (%)		0					0			
P value		0.8590					0.7131			
Medial gastrocnemius										
Mean (SD)	1.19 (0.08)		1.19 (0.05)		–	1.21(0.07)		1.19 (0.07)		–
∆ (%)		0					− 1			
P value		0.8240					0.2078			
Lateral gastrocnemius										
Mean (SD)	1.10 (0.10)		1.08 (0.07)		–	1.09 (0.08)		1.09 (0.06)		–
∆ (%)		− 2					0			
P value		0.2676					0.7558			
Soleus										
Mean (SD)	1.19 (0.09)		1.20 (0.06)		–	1.20 (0.08)		1.20 (0.08)		–
∆ (%)		+1					0			
P value		0.4045					0.8302			
Extensors										
Mean (SD)	1.22 (0.10)		1.24 (0.10)		–	1.21 (0.07)		1.23 (0.08)		–
∆ (%)		+2					+1			
P value		0.1345					0.5989			

*ME/CFS* Myalgic Encephalomyelitis/Chronic Fatigue Syndrome. Mean values of sodium and water content with standard deviation (SD) in brackets. ∆ (%) is percentage of increase/decrease after exercise and after recovery period of 40 minutes. P value refers to comparison of baseline values with values directly after exercise and values directly after exercise with values after 40 minutes recovery, respectively

### Comparison of tissue sodium content in patients with ME/CFS and controls

Baseline tissue sodium content was higher in all 5 muscle compartments in ME/CFS compared to controls (Fig. 2). The strongest difference was observed within the anterior extensor muscle compartment, with 12.2 ± 1.66 mM in patients and 9.38 ± 0.71 mM in controls (p = 0.0034). After exercise tissue sodium content increased in all exercised muscles and was higher in ME/CFS than in controls at all 8 time points during the 40-min measurement period (p < 0.0001, respectively). The decrease of sodium content in medial gastrocnemius was slower in ME/CFS than in controls with + 30% versus + 17% to baseline at 12 min (p = 0.0326) and + 29% versus + 16% to baseline at 15 min (p = 0.0265). No difference in tissue water content between the two groups was detected in any muscle compartment at any point in time.

### Handgrip strength and correlation with tissue sodium content

**Fig. 4:**
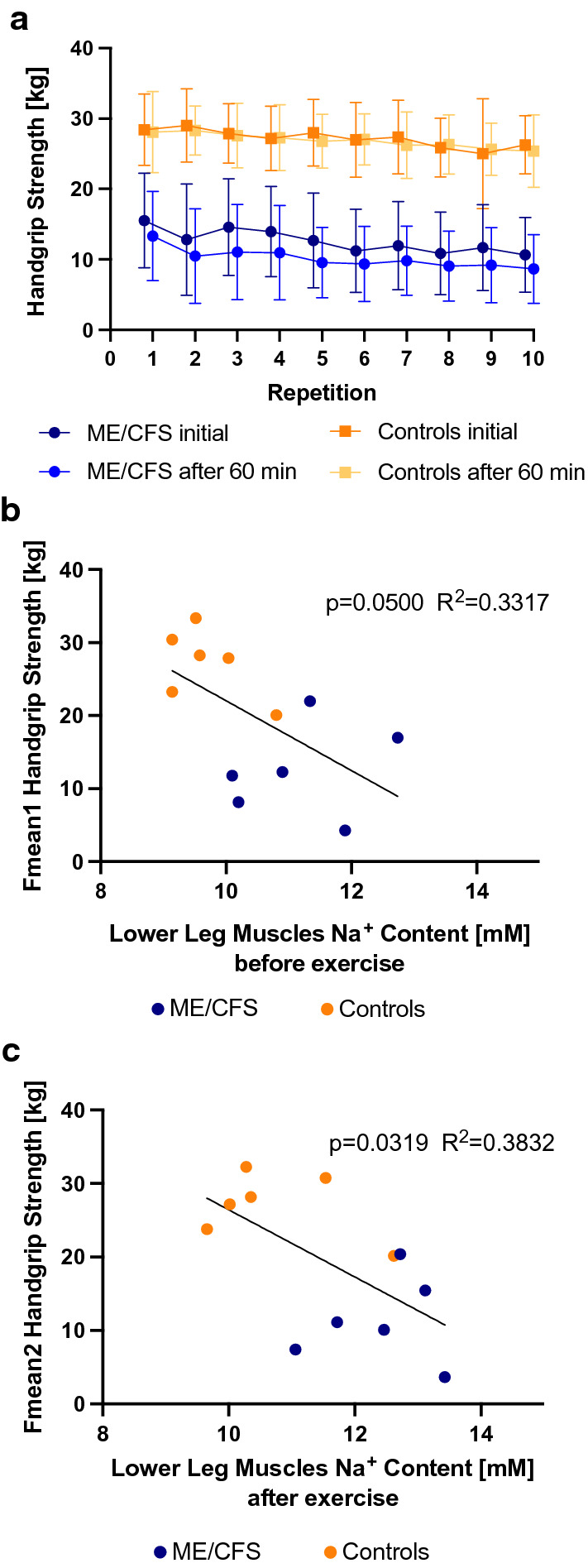
**a** Handgrip strength measurement with 10 repeats in two sessions. Mean values in kg with standard deviation. Black line shows results of initial session and grey line of second session after 60 min. ME/CFS patients show lower mean handgrip strength compared to controls and perform worse in their second session. Mean handgrip strength of controls remains unchanged after 60 min. The scatter plot with linear regression line shows (**b**) an inverse correlation of baseline tissue sodium content in lower leg muscles (average of triceps, extensors, medial and lateral gastrocnemius and soleus) and initial mean handgrip strength (p = 0.0500, R^2^ = 0.3317, N = 12) and **c** an inverse correlation of average tissue sodium content in lower leg muscles after exercise (minutes 1–40) and mean handgrip strength of the second session (p = 0.0319, R^2^ = 0.3832, N = 12)

Patients with ME/CFS showed reduced maximal and mean handgrip strength compared to controls (fmean1 12.6 ± 6.3 kg in patients versus 27.2 ± 4.8 kg in controls; p = 0.0011) and after 60 min (fmean2 10.1 ± 5.6 in patients versus 26.9 ± 4.2 in controls; p = 0.0002). Further, the recovery rate fmean2/fmean1 in patients was lower (p = 0.0055), while controls showed similar mean handgrip strength in both sessions (p = 0.5342, Fig. 4a). We observed an inverse correlation between fmean1 handgrip strength and baseline tissue sodium content of all lower leg muscles (average of triceps, extensors, medial and lateral gastrocnemius and soleus; p = 0.0500, R^2^ = 0.3317, Fig. 4b), and between fmean2 handgrip strength and average tissue sodium content of all lower leg muscles after exercise (p = 0.0319, R^2^ = 0.3832, N = 12, Fig. 4c). The correlations of handgrip strength and the individual muscles are shown in Additional file 1: Fig. S1.

## Discussion

To the best of our knowledge, we conducted the first ^23^Na-MRI study in ME/CFS. This study provides evidence that sodium content of lower leg muscles is higher in ME/CFS than in healthy controls at rest and after exercise. Furthermore, our findings indicate an inverse correlation between mean muscle sodium content and handgrip strength. Thus, sodium overload may play a role in the pathophysiology of ME/CFS and may allow for potential treatment targeting.

This study demonstrates the feasibility of monitoring changes in muscle sodium content in ME/CFS and healthy subjects after exercise using ^23^Na-MRI. As already shown in previous studies in healthy persons, our study populations also had an increase in muscle tissue sodium content directly after anaerobic exercise [27, 29]. Our MR images indicate an increase of sodium without concomitant water increase, as it was shown in another study, which postulates that sodium can be stored non-osmotically [19]. Our findings that tissue sodium content in healthy controls returned to baseline levels approximately 40 min after muscle exercise correspond to the results of a study showing a return to baseline levels 35 min after exercise in healthy controls [30]. Like healthy subjects, patients with ME/CFS responded to exercise with an initial increase in muscle sodium content followed by a decrease over time. Since all patients with ME/CFS suffered from post-exertional malaise after exercise, we would not recommend this examination method for clinical routine as long as no implications the diagnostic or therapeutic management of the patients can be made. Nevertheless, the burden of the disease is high, and the identification of potential treatment targets may merit further scientific use of exercise testing in conjunction with the non-invasive MRI method.

**Fig. 5:**
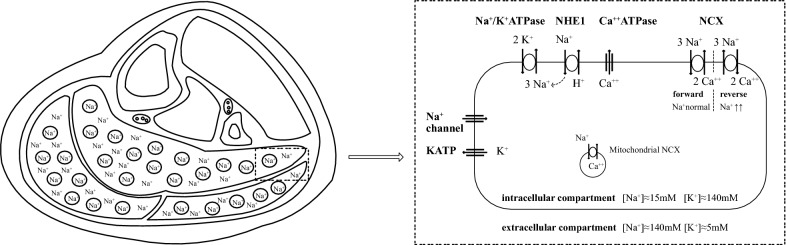
Illustration of the lower leg cross-section with exemplary representation of intracellular and extracellular sodium in the muscle tissue. The zoomed section on the right shows a myocyte with all its channels and transporters involved in the handling of protons, sodium, calcium, and potassium. Physiologically, the NCX works in forward mode to export calcium. High sodium concentration caused by high NHE1- and low Na+/K+-ATPase activity can reverse the transport direction of the NCX to import calcium instead of exporting it, resulting in calcium overload

The findings of our study are in line with our recent hypothesis paper on the mechanisms of the energetic situation in muscles in ME/CFS and the underlying disturbance in ion homeostasis [14]. Appropriate muscular perfusion as well as function of the Na+/K+-ATPase determine muscle fatigability. The sodium-proton exchanger subtype1 (NHE1) exports protons via the import of sodium ions. In poor energetic situations increased proton production raises intracellular sodium via NHE1, the most important proton-extruder in skeletal muscle. Endothelial dysfunction leads to muscle hypoperfusion and diminished ATP generation in ME/CFS [9]. Sodium is removed from the muscle by the Na+/K+-ATPase at the expense of ATP consumption. We assume that the removal of sodium is further impaired due to dysfunction of the ß2 adrenergic receptor which leads to an insufficient stimulation of the Na+/K+-ATPase [14]. High intracellular sodium can reverse the transport direction of the sodium–calcium exchanger (NCX) to import calcium instead of exporting which is also known from the ischemia–reperfusion paradigm [38]. Channels and transporters that play a role in ion transport in myocytes are depicted in Fig. 5. The ensuing calcium overload affects the mitochondrial metabolism and the endothelium, which further worsens the energetic situation in a vicious circle which can explain post-exertional malaise, exercise intolerance and chronification. Changes in intracellular and mitochondrial calcium via NCX induced by the rise in intramuscular sodium are considered the key pathomechanism in the energetic and mitochondrial disturbance in ME/CFS as outlined in a recent hypothesis paper [9, 14] but cannot be directly demonstrated with current methods in vivo. The demonstration of elevated intramuscular sodium in this study provides, however, evidence that the conditions for a disturbed calcium handling via the NCX are indeed present in skeletal muscles in ME/CFS. The biological significance of these results is a better understanding of the pathophysiology of ME/CFS. This is a prerequisite for developing therapeutic strategies for this frequent and debilitating disease for which no effective treatment exists so far.

The Na+/K+-ATPase exchanges three sodium ions for two potassium ions. Insufficient stimulation is expected to lead to a rise in intracellular sodium and concomitantly to a decrease in intracellular potassium. Here we show a rise in intracellular sodium post-exercise. Concerning potassium, there is evidence for a decrease in intracellular potassium from two publications. Reduced potassium efflux was found in exercising muscle in ME/CFS patients which can only be explained by the development of intracellular hypokalemia [39]. The authors incriminated ROS to inhibit the Na+/K+-ATPase. In an earlier paper total body potassium was found decreased by about 10% in a group of ME/CFS patients with severe fatigue [40]. In this study, there was a strong inverse correlation of the total body potassium and the total time spent resting as a measure of fatigue and exhaustion. Lower plasma potassium levels were also found in a recent study in patients with ME/CFS compared with healthy controls [41]. If potassium leaving the cell during the process of repolarization via potassium channels is not taken up fast enough by the Na^+^/K^+^-ATPase in the working muscles, it reaches the blood stream to be renally excreted so that loss of potassium occurs during exercise. Thus, the indirect evidence for a lowered intracellular potassium together with our direct demonstration of a rise in intracellular sodium provides very strong arguments for a diminished Na+/K+-ATPase activity in ME/CFS.

Since it is technically not possible to measure intracellular sodium during exercise the possibility exists that sodium rises much higher than we measured pre- and post-exercise. During exercise sodium loading must be higher compared with rest because of sodium entry via sodium channels in the process of excitation which adds to sodium import via ion transporters of whom NHE1 may be the most important one. If this is a very dynamic process there may be a rise of sodium during exercise (reaching a steady state) over the level we see post-exercise, but sodium does not necessarily accumulate to a level that it would take minutes to remove the excess. The rise in sodium we found post-exercise may be due to increased NHE1-activity post-exercise due to proton extrusion as a consequence of a glycolytic metabolism in the presence of a diminished Na+/K+-ATPase activity. Jones et al. reported a decreased proton extrusion immediately post-exercise [13]. It is possible that NHE1 activity is diminished during exercise due to high intracellular sodium reducing its driving force. This is even the only plausible explanation for the observed reduced proton extrusion. It is also an argument for a higher sodium rise during exercise compared with post-exercise. Immediately after exercise intracellular sodium can quickly fall as there is no more sodium entry via sodium channels to restore the driving force of the NHE1. Protons can indeed accumulate during exercise (intracellular acidosis). Removing the excess via the NHE1 (after restoring its driving force) at rest could then cause the rise in intracellular sodium we found post-exercise.

The intensity values of the 4 reference phantoms with different NaCl solutions allowed us to measure sodium content via the signal intensities. After translating intensity to concentration in a linear trend analysis, we used mM as the unit to express tissue sodium content. However, it should be noted that with the methods of our scan protocol the signal decays very quickly and parts of tissue sodium are no longer visible and measurable at the time of measurement. Therefore, our values in mM are lower than those from previous publications and cannot be directly compared to them.

We measured total sodium content in muscle tissue and small vessels which supply muscle tissue. Long- and short-lived sodium components are measurable via ^23^Na-MRI. The long-lived components are assumed to be the mobile, liquid sodium, which is mainly extracellular sodium and short-lived sodium is assumed to be intracellular sodium. Since only about 30% of the short-lived sodium is still visible at TE 2.44, which we used in our protocol, we assume that we measured mainly the long-lived sodium, since a large number of short-lived sodium signals have already decayed [34]. Like Hammon et al. we focused on postexercise imaging results, which would not have been possible with scan protocols depicting more intracellular sodium, because they take longer than 10 min [27]. Within this period, however, we already observed a decrease in sodium content. For exact differentiation between intracellular and extracellular sodium, we recommend the realization of further studies with radial sequences and shorter TEs. Since the MRI method used has a potential clinical benefit, optimization of the technique for applications in larger clinical cohorts or even clinical routine would be desirable. To make this possible, we believe that one of the most important features would be the reduction of motion artefacts through e.g. navigator pulses or camera control.

## Limitations

Since our study was a preliminary study, it was performed with a very small sample. In our pilot study we found despite the small size significant differences between ME/CFS and healthy controls and showed in the results the ranges and standard deviations as well as the individual measurements in the scatter plot with the linear regression line (Fig. 4). This information will be valuable for planning future studies on larger cohorts, possibly with therapeutic interventions.

Moreover, it is unclear to what extent the immobility of patients with ME/CFS has influenced the results. It is possible that results were affected by variable exercise intensity, muscle condition and activation. This potential bias could be prevented in further studies by using a purpose-built, MRI-compatible exercise apparatus for calf muscles. However, we established our exercise protocol after extensive literature review and internal tests, and we assume that sufficient load matters more than the type of exercise. The same exercise protocol was followed in all patients. The exercise was performed for 3 min to exhaustion and checked for correctness of performance and intensity by a person of the researchers’ team. The fact that all 12 study participants experienced muscle soreness or muscle pain after the exercise is also an indicator for a sufficient muscle load.

All data were processed and analyzed in Horos by one person. Previous analyses of inter- and intra-operator variability in ROI-based measurements, however, revealed no significant differences between measurements [17, 37, 42].

## Conclusion and outlook

^23^Na-MRI confirms our hypothesis of increased sodium content in muscles of ME/CFS patients. It provides the opportunity to study the sodium homeostasis in patients with ME/CFS. Our findings lead to a better understanding of the pathophysiology of ME/CFS and open up diagnostic possibilities and potential therapeutic targeting. As our results are encouraging, further research with a larger study population and an adapted methodology should be conducted.

## Supplementary Information

**Additional file 1: Figure S1.** Correlations of handgrip strength and tissue sodium content.

## Data Availability

All data generated or analyzed during this study are included in this published article and its supplementary information files.

## Abbreviations

ANCOVA: Analysis of covariance
A.U.: Arbitrary units
BMI: Body mass index
CFS: Chronic Fatigue Syndrome
DICOM: Digital imaging and communications in medicine
EBV: Epstein-Barr virus
FA: Flip angle
ME: Myalgic Encephalomyelitis
mM: Millimolar
MRI: Magnetic resonance imaging
Na: Sodium
NaCl: Sodium chloride
ROI: Region of Interest
SARS-Cov2: Severe acute respiratory syndrome coronavirus type 2
SD: Standard deviation
SE: Spin echo
TA: Scan duration
TE: Echo times
TI: Inversion time
TR: Repetition time
T2: Transversal relaxation time
