# Correction: Muscle sodium content in patients with Myalgic Encephalomyelitis/Chronic Fatigue Syndrome

**DOI:** 10.1186/s12967-023-03948-4

**Published:** 2023-04-04

**Authors:** Elisabeth Petter, Carmen Scheibenbogen, Peter Linz, Christian Stehning, Klaus Wirth, Titus Kuehne, Marcus Kelm

**Affiliations:** 1 Institute of Medical Immunology, Charité Universitätsmedizin Berlin, Campus Virchow, Berlin, Germany; 2 Institute of Computer-Assisted Cardiovascular Medicine, Charité Universitätsmedizin Berlin, Berlin, Germany; 3 Department of Congenital Heart Disease, German Heart Center Berlin, Berlin, Germany; 4 Institute of Radiology, Friedrich‐Alexander‐Universität Erlangen‐Nürnberg (FAU), University Hospital Erlangen, Erlangen, Germany; 5 Philips Healthcare, Hamburg, Germany; 6 Institute of General Pharmacology and Toxicology, University Hospital Frankfurt Am Main, Goethe-University, Theodor-Stern Kai 7, Frankfurt am Main, Germany; 7 Berlin Institute of Health (BIH), Berlin, Germany; 8 German Centre for Cardiovascular Research (DZHK), Partner Site, Berlin, Germany

**Correction** **:** **Journal of Translational Medicine (2022) 20:580** 10.1186/s12967-022-03616-z

Prior to publication of the original article [1], we have been notified that Table 2 formatting was incorrectly set up and ORCIDs were missing. Instead of a finalized document, a preliminary version had been published without authors approval with an unnecessary red line under Table 2 that should be removed. Authors should also have their ORCIDs assigned in this correction:

- Carmen Scheibenbogen https://orcid.org/0000-0002-8554-9638
- Titus Kuehne https://orcid.org/0000-0003-1631-4824
- Peter Linz https://orcid.org/0000-0002-8232-1443
- Christian Stehning https://orcid.org/0000-0002-0660-840X
- Elisabeth Petter https://orcid.org/0000-0002-0285-2851
- Klaus Wirth https://orcid.org/0000-0001-9862-4951

**Table 2:** Tissue sodium content and tissue water content

	ME/CFS					Controls				
	Baseline		Directly after exercise		40 minutes after exercise	Baseline		Directly after exercise		40 minutes after exercise
Na^+^ [mM]										
Triceps surae										
Mean (SD)	10.83 (1.15)		12.77 (0.90)		11.43 (1.51)	9.93 (0.79)		11.58 (1.13)		10.33 (1.12)
∆ (%)		+18		− 10			+17		− 11	
P value		<0.0001		0.0090			0.0081		0.0002	
Medial gastrocnemius										
Mean (SD)	10.50 (0.78)		13.65(0.69)		11.67 (1.30)	10.23 (1.24)		12.67 (1.28)		10.75 (1.11)
∆ (%)		+30		− 15			+24		− 15	
P value		0.0005		0.0182			0.0007		0.0002	
Lateral gastrocnemius										
Mean (SD)	11.60 (1.58)		13.85 (2.01)		11.83 (1.54)	9.33 (0.82)		11.67 (2.01)		9.65 (1.44)
∆ (%)		+19		− 15			+25		− 17	
P value		0.0060		0.0023			0.0373		0.0099	
Soleus										
Mean (SD)	10.85 (1.58)		11.58 (1.34)		10.95 (1.59)	9.63 (0.58)		10.33 (1.56)		9.60 (0.89)
∆ (%)		+7		− 5			+7		− 7	
P value		0.0197		0.1334			0.3220		0.0974	
Extensors										
Mean (SD)	12.20 (1.66)		11.87 (1.37)		11.77 (1.81)	9.38 (0.71)		10.10 (1.28)		9.97 (1.45)
∆ (%)		− 3		− 1			+8		− 1	
P value		0.5354		0.9014			0.2492		0.7796	
H_2_O [kg/L]										
Triceps surae										
Mean (SD)	1.16 (0.09)		1.16 (0.06)		–	1.16(0.07)		1.16 (0.07)		–
∆ (%)		0					0			
P value		0.8590					0.7131			
Medial gastrocnemius										
Mean (SD)	1.19 (0.08)		1.19 (0.05)		–	1.21(0.07)		1.19 (0.07)		–
∆ (%)		0					− 1			
P value		0.8240					0.2078			
Lateral gastrocnemius										
Mean (SD)	1.10 (0.10)		1.08 (0.07)		–	1.09 (0.08)		1.09 (0.06)		–
∆ (%)		− 2					0			
P value		0.2676					0.7558			
Soleus										
Mean (SD)	1.19 (0.09)		1.20 (0.06)		–	1.20 (0.08)		1.20 (0.08)		–
∆ (%)		+1					0			
P value		0.4045					0.8302			
Extensors										
Mean (SD)	1.22 (0.10)		1.24 (0.10)		–	1.21 (0.07)		1.23 (0.08)		–
∆ (%)		+2					+1			
P value		0.1345					0.5989			

*ME/CFS* Myalgic Encephalomyelitis/Chronic Fatigue Syndrome. Mean values of sodium and water content with standard deviation (SD) in brackets. ∆ (%) is percentage of increase/decrease after exercise and after recovery period of 40 minutes. P value refers to comparison of baseline values with values directly after exercise and values directly after exercise with values after 40 minutes recovery, respectively
