# Supplementary material for: Muscle sodium content in patients with Myalgic Encephalomyelitis/Chronic Fatigue Syndrome
Source: J Transl Med. 2022 Dec 9;20:580. doi: 10.1186/s12967-022-03616-z (PMC9733289; doi:10.1186/s12967-022-03616-z)
Supplement: Supplementary file 1 — Additional file 1: Figure S1. Correlations of handgrip strength and tissue sodium content. [file 12967_2022_3616_MOESM1_ESM.docx]

Supplementary Figure S1. Correlations of Handgrip Strength and Tissue Sodium Content

*Figure S1. Correlation of Handgrip Strength and Tissue Sodium Content of the Individual Muscles. Scatter blot combined with linear regression line of Pearson correlation coefficient. Left column shows*

*baseline tissue sodium content in lower leg muscles (average of triceps, extensors, medial and lateral gastrocnemius and soleus) and initial mean handgrip strength (fmean1), and right column shows average tissue sodium content in lower leg muscles after exercise (minute 1-40) and mean handgrip strength of the second session (fmean2). A tendency towards an inverse correlation between handgrip strength and tissue sodium content is evident, especially in the triceps surae muscle (p=0.0203) and medial gastrocnemius muscle (p=0.0095) after exercise.*
